# Pathogenic variant profile in DNA damage response genes correlates with metastatic breast cancer progression-free survival in a Mexican-mestizo population

**DOI:** 10.3389/fonc.2023.1146008

**Published:** 2023-04-27

**Authors:** Rafael Vázquez-Romo, Oliver Millan-Catalan, Erika Ruíz-García, Antonio D. Martínez-Gutiérrez, Alberto Alvarado-Miranda, Alma D. Campos-Parra, César López-Camarillo, Nadia Jacobo-Herrera, Eduardo López-Urrutia, Mariano Guardado-Estrada, David Cantú de León, Carlos Pérez-Plasencia

**Affiliations:** ^1^ Departamento de Cirugía de Tumores Mamarios, Instituto Nacional de Cancerología (INCan), Ciudad de México, Mexico; ^2^ Laboratorio de Genómica, Instituto Nacional de Cancerología (INCan), Ciudad de México, Mexico; ^3^ Laboratorio de Medicina Traslacional y Departamento de Tumores Gastrointestinales, Instituto Nacional de Cancerología, CDMX, Mexico; ^4^ Dirección de Investigación, Instituto Nacional de Cancerología (INCan), Ciudad de México, Mexico; ^5^ Posgrado en Ciencias Genómicas, Universidad Autónoma de la Ciudad de México, Ciudad de México, Mexico; ^6^ Unidad de Bioquímica, Instituto Nacional de Ciencias Médicas y Nutrición, Salvador Zubirán (INCMNSZ), Ciudad de México, Mexico; ^7^ Laboratorio de Genómica, Unidad de Biomedicina, FES-IZTACALA, UNAM, Tlalnepantla, Mexico; ^8^ Laboratorio de Genética, Ciencia Forense, Facultad de Medicina, Universidad Nacional Autónoma de México, Ciudad de México, Mexico

**Keywords:** metastatic breast cancer, DNA damage response, Latin American population, Mexican-mestizo population, Progression free survival (PFS)

## Abstract

**Introduction:**

Metastatic breast cancer causes the most breast cancer-related deaths around the world, especially in countries where breast cancer is detected late into its development. Genetic testing for cancer susceptibility started with the BRCA 1 and 2 genes. Still, recent research has shown that variations in other members of the DNA damage response (DDR) are also associated with elevated cancer risk, opening new opportunities for enhanced genetic testing strategies.

**Methods:**

We sequenced BRCA1/2 and twelve other DDR genes from a Mexican-mestizo population of 40 metastatic breast cancer patients through semiconductor sequencing.

**Results:**

Overall, we found 22 variants –9 of them reported for the first time– and a strikingly high proportion of variations in ARID1A. The presence of at least one variant in the ARID1A, BRCA1, BRCA2, or FANCA genes was associated with worse progression-free survival and overall survival in our patient cohort.

**Discussion:**

Our results reflected the unique characteristics of the Mexican-mestizo population as the proportion of variants we found differed from that of other global populations. Based on these findings, we suggest routine screening for variants in ARID1A along with BRCA1/2 in breast cancer patients from the Mexican-mestizo population.

## 1 Introduction

As the number of new breast cancer cases and fatalities continues to rise worldwide (1), detection and treatment of this disease is more of a pressing issue for researchers and health professionals. Widely adopted screening programs have proven efficient at detecting stage I-II cases before they develop further into stage III and compromise survival, effectively decreasing the fatal cases. Yet, a significant amount of these deaths occur in stage IV or metastatic patients (2, 3).

An important aspect of the screening programs that has become increasingly widespread with the advancement of technology is genetic testing for cancer susceptibility. The first surveyed genes were *BRCA 1* and *2*, where germline variants are associated with around 25% of breast cancer cases (4). But recent research has revealed that several other genes that also participate in the DNA damage response (DDR) also confer increased breast cancer risk, such as *PALB2, TP53, RAD50, RAD51D*, and *CHEK2*, among others (5, 6). For example, variants in *PALB2* (Partner and Localizer of *BRCA2*) are associated with an estimated cumulative risk of breast cancer of 14% (7). Somatic alterations, on the other hand, are associated with high-grade tumor progression; for instance, *TP53* variants correlate with metastasis spread and relapse risk (8, 9). Both germline and somatic alterations drive tumor development cooperatively and influence response to treatment (10, 11).

Variants in the DNA damage response (DDR) genes –a complex machinery encompassing several pathways that maintain the integrity of DNA within the cell (12)– produce malfunctioning proteins that restrict the ability of cells to repair DNA lesions, rendering them susceptible to genetic instability and cancer development (13); such is the case of *ARID1A*, a recently studied subunit of the SWI/SNF chromatin remodeler complex whose variants have been associated with breast cancer brain metastasis (14).The presence of these variants also influences treatment choice, as patients carrying them can benefit from treatment alternatives that target the DDR to create synthetic lethality by inhibiting the Poly ADP-ribose (PARP) –a polymerase that synthesizes DNA in the final steps of the repair process– with purpose-designed drugs (15, 16).

Our group is interested in studying the distribution of BRCA variants, particularly in the Mexican-mestizo population, where we have found unreported variants (17, 18), confirming that variation distribution can vary significantly from one geographical location to another (19, 20). So, surveying local populations looking for characteristic individual variations or patterns is an important stepping stone toward universal tailored diagnostics and treatments, especially in Latin American countries where breast cancer is mostly diagnosed in later stages (21).

In this work, we sequenced fourteen DDR genes in a Mexican cohort of 40 metastatic breast cancer patients, searching for an association between variants in DDR genes and response to treatment. The genes we sequenced belong mainly to the DNA Interstrand Crosslink Repair, a DDR pathway that has been recently associated which high tumor burden in breast carcinomas (22); the rest were DNA damage sensors (*ATM, CHK2*) and transcriptional activators (*ARID1A, TP53*) that had been recently associated with metastatic breast cancer (23–25). We found 19 unique variants, from which 9 had not been reported before, and a correlation between the combination of alterations in the *ARID1A, BRCA1, BRCA2*, and *FANCA* genes and progression-free survival. To our knowledge, the association between alterations in DDR genes other than BRCA and treatment response in metastatic breast cancer had not been surveyed yet in the Mexican-mestizo population.

## 2 Materials and methods

### 2.1 Patient cohort

This study included prospectively tumor biopsy and clinical data from forty stage-IV breast cancer patients that attended the Instituto Nacional de Cancerología (INCan, Mexico City, Mexico. The study was approved by INCan’s Review Board and Ethics Committee (016/010/IBI; CEI/1001/16); all patients signed informed consent. After the surgical excision, tumor biopsies were segmented into two pieces, one for pathological confirmation and another for DNA extraction.

### 2.2 Patients and clinical outcome assessment

A total of 40 patients were enrolled diagnosed with metastatic breast cancer confirmed by positron emission tomography (PET) and computed tomography (CT) scans. All patients were treated according to the National Comprehensive Cancer Network (NCCN) guidelines. Clinical outcome was evaluated by The Response Evaluation Criteria in Solid Tumors (RECIST Version 1.1) at baseline and at 6 months (26). Progression-free survival (PFS) was defined as the time from the commencement of treatment until disease progression or the last visit. Overall survival (OS) was defined as the time from diagnosis until death or the last visit.

### 2.3 DNA extraction

Tumor DNA was extracted using the QIAamp DNA Blood Mini kit (Qiagen, cat. no. 51106). following the manufacturer’s recommendations. DNA integrity was verified by agarose electrophoresis and the concentration was determined using RNase P Detection Reagent (FAM) (Applied Biosystems, cat. no. 4316831).

### 2.4 Targeted sequencing

Fourteen DDR genes were selected for sequencing. Two of them, *BRCA1* and *BRCA2*, were amplified using Ion Ampliseq *BRCA 1* and *2* panel (Thermo Fisher Scientific); this panel includes 167 primers pairs in three pools. The remaining genes, *ARID1A, ATM, CHK2, FANCA, FANCB, FANCC, FANCD2, PARP1, PALB2, RAD50, RAD51*, and *TP53* were amplified with the custom panel IAD94476_197_Designed; this panel includes 440 primers in two pools. (**Supplementary Table 1**). Libraries were prepared from 25 ng DNA, and amplification of each patient’s DDR-genes was identified using a unique Ion Xpress barcode adapter (Thermo Fisher Scientific cat. no. 4471250). For sequencing, we used the Ion PGM Hi-Q Sequencing kit (REFA25589) in the Ion torrent PGM (Personal Genome Machine) instrument (Thermo Fisher Scientific).

### 2.5 Data analysis

The sequences were aligned to the hg19 human reference genome (GRCh37). The.bam files were exported to the Ion Reporter version 5.18 for variation analysis. Pathogenic and probably pathogenic variants were classified according to the American College of Medical Genetics and Genomics guidelines (27)

Kaplan–Meier curves and Cox regressions were calculated using the survival package in R (R version 4.2.2, we used the survival package version 3.4.0.). Variable selection for the Multivariate Cox regressions was performed using a forward stepwise procedure. Statistical significance was defined as p <0.05 (two sided).

## 3 Results

**Figure 1:**
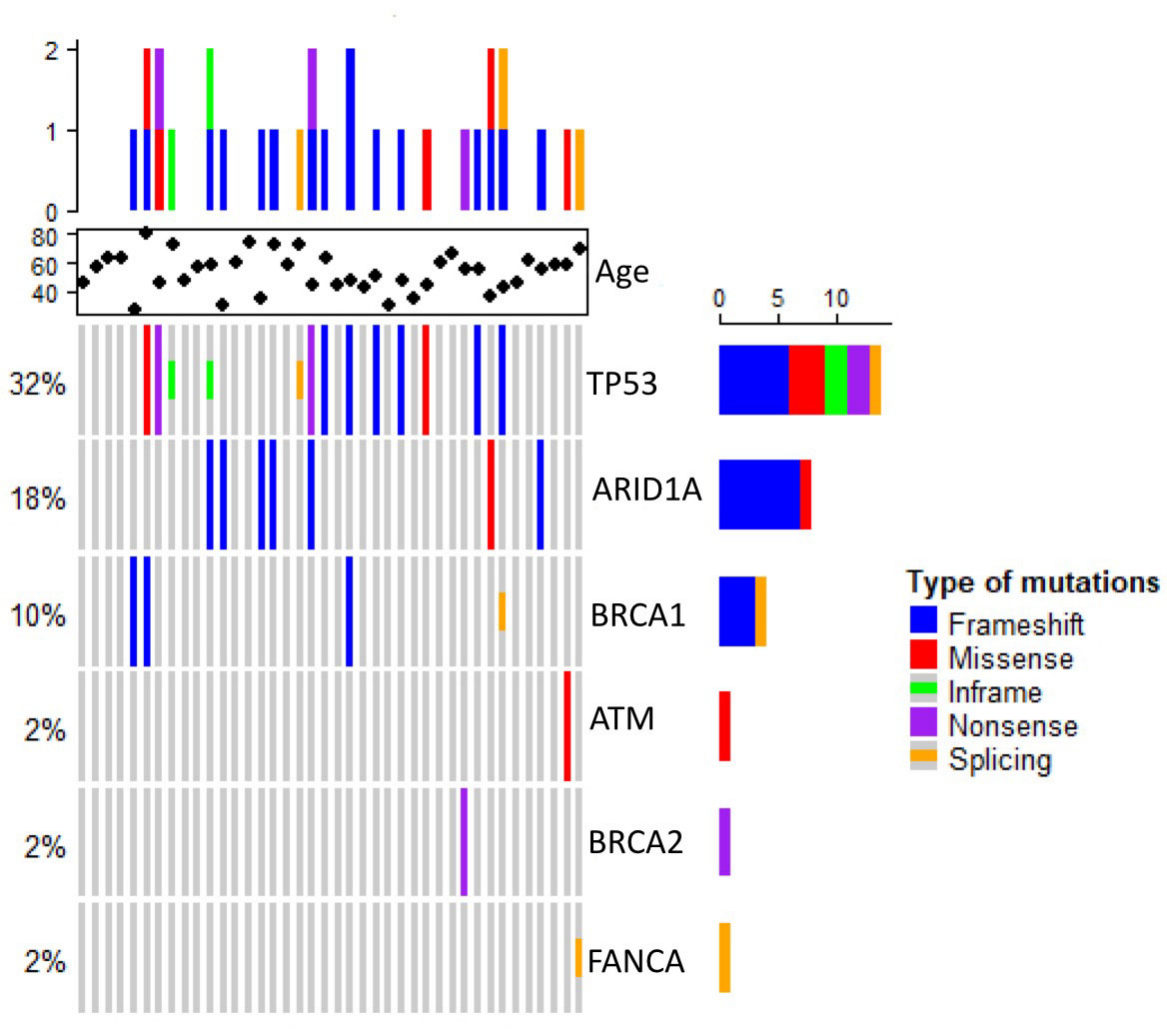
Heat map showing somatic variants profiles of metastatic breast cancer.

The 40 stage-IV screened tumor samples came from patients with a mean age of 53 years, ranging from 27 to 81 (n = 40). Most tumors were ductal (85%) and the remainder, lobular (15%). The molecular type corresponded with previously reported proportions; Luminal A and B tumors were more frequent that HER+ and TNBC. Notably, 16 of the 40 samples came from patients with a family history of cancer (**Table 1**). Twenty-two of the samples carried at least one variant in the sequenced genes, one had multiple variants in BRCA1, two had multiple variants in TP53, and two had variants in two genes –*ARID1A* and *ATM* or *FANCA* and *TP53* (**Figure 1**, **Table 2**)

**Table 1:** Clinical characteristics of 40 unrelated metastatic breast cancer patients.

Characteristics	No. (%) (n= 40)
Age, mean (range, years)	53 (27-81)
Menopausal status	
Premenopausal	18 (45)
Postmenopausal	22 (55)
Histology	
Ductal	34 (85)
Lobular	6 (15)
Tumor size	
TX	1 (2.5)
T1	1 (2.5)
T2	4 (10)
T3	7 (17.5)
T4	27 (67.5)
Nodes	
N1	8 (20)
N2	8 (20)
N3	24 (60)
Molecular subtype	
Luminal A	18 (45)
Luminal B	10 (25)
HER+	5 (12.5)
TNBC	7 (17.5)
Cancer family history	
Breast/Ovarian cancer	6 (15)
Other cancers	10 (25)
No	24 (60)
Chemotherapy regime	
Antimitotic	14 (35)
Hormone therapy	12 (30)
Alkylating-antimitotic	10 (25)
Alkylating	4 (10)
Status	
Alive	13 (32.5)
Dead	27 (67.5)

Numbers in parenthesis express percentages.

**Table 2:** Genes with variants in unrelated metastatic breast cancer samples.

ARID1A	ATM	BRCA1	BRCA2	FANCA	TP53
M11	M15	M05 (3)	M31	M42	M06 (2)
M12	M33	M34			M08
M15	M40				M18 (2)
M16					M20 (3)
M19					M22
M38					M24
					M26
					M28
					M32
					M42

Numbers in parentheses indicate the different variations present in the samples.

Overall, we found 19 unique sequence variants in the 22 samples carrying them, 10 had been already identified and 9 were previously unreported. Ten (52%) of the found variants were in the TP53 gene. The most frequent alterations in this gene –c.742C>T and c.215_216insG– were only the second most frequent, while the first was c.3980_3981insC, in ARID1A. Thirteen variants were found only once in the studied population. Strikingly, no variants in the *CHK2, FANCB, FANCC, FANCD2, PALB2, RAD50*, or *RAD51* genes were present in our cohort (**Figure 1**, **Table 3**). Pathogenic or likely pathogenic variants are depicted in **Supplementary Table 1**.

**Table 3:** Variants found in 40 unrelated metastatic breast cancer samples.

Gene	Coding Sequence Position	AminoAcid Change	Significance*	dbSNP	Frequency
ARID1A	c.3977_3980delCGCA	p.Pro1326ArgfsTer154	LP	not reported	2
ARID1A	c.3980_3981insC	p.Gln1327HisfsTer11	LP	not reported	4
ATM	c.6861delA	p.Val2288SerfsTer22	LP	not reported	1
ATM	c.8124T>A	p.Asp2708Glu	LP	rs587781990	2
BRCA1	c.3759_3760delTA	p.Lys1254GlufsTer12	P	rs80357520	1
BRCA1	c.5054_5060delCTCATGT	p.Thr1685MetfsTer3	LP	not reported	2
BRCA1	c.5277 + 1delG	splice site	P	rs273901754	1
BRCA2	c.5635G>T	p.Glu1879Ter	P	rs55996097	1
FANCA	c.709 + 5G>A	splice site	P	rs759877008	1
TP53	c.1123C>T	p.Gln375Ter	LP	rs1555524156	1
TP53	c.215_216insG	p.Val73ArgfsTer76	LP	not reported	3
TP53	c.522_539delGCGCTGCCCCCACCATGA	p.Pro177_Cys182del	P	not reported	1
TP53	c.560-1G>A	splice site	P	rs1202793339	1
TP53	c.586C>T	p.Arg196Ter	P	rs397516435	1
TP53	c.626_627delGA	p.Arg209LysfsTer6	P	rs1057517840	1
TP53	c.718delA	p.Ser240ValfsTer7	LP	not reported	1
TP53	c.742C>T	p.Arg248Trp	P	rs121912651	3
TP53	c.815_817dup	p.Val272_Arg273insLeu	P	not reported	1
TP53	c.866_873delTCCGCAAG	p.Leu289GlnfsTer14	LP	not reported	1

*Clinical Significance according to stablished criteria [24]. LP, likely pathogenic; P, pathogenic.

**Figure 2:**
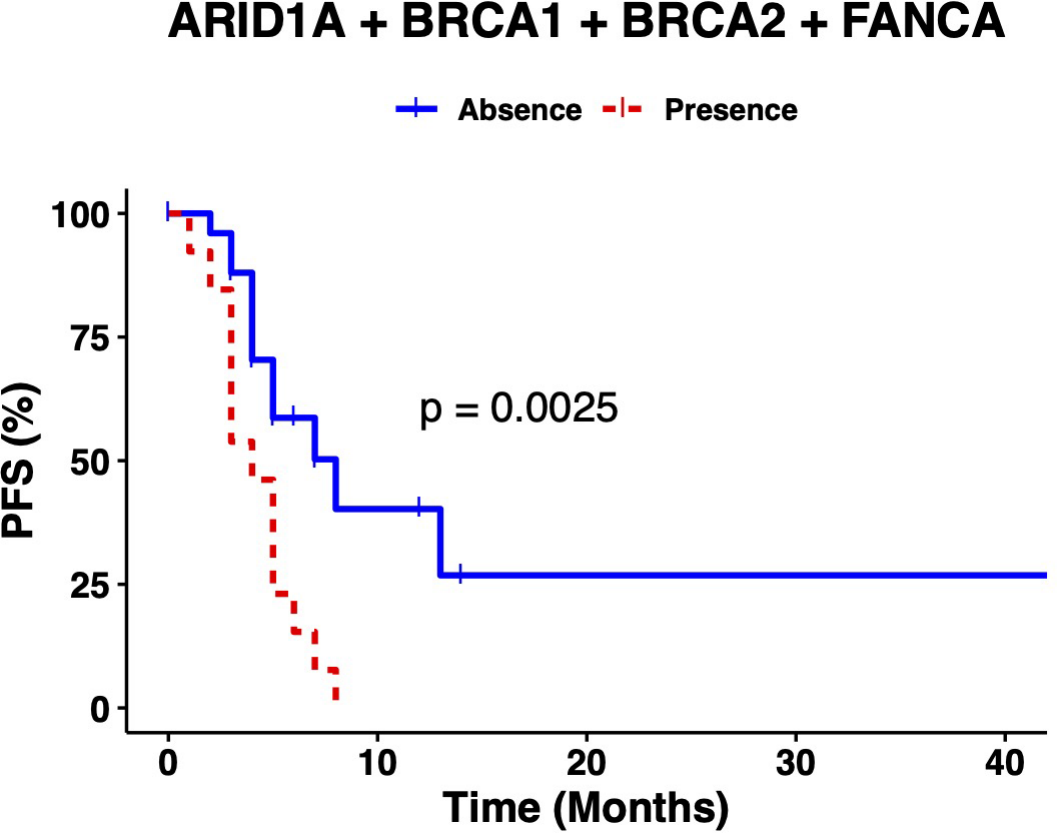
The presence of at least one variant in *ARID1A*, *BRCA1*, *BRCA2 or FANCA* genes correlates with lower PFS.

**Figure 3:**
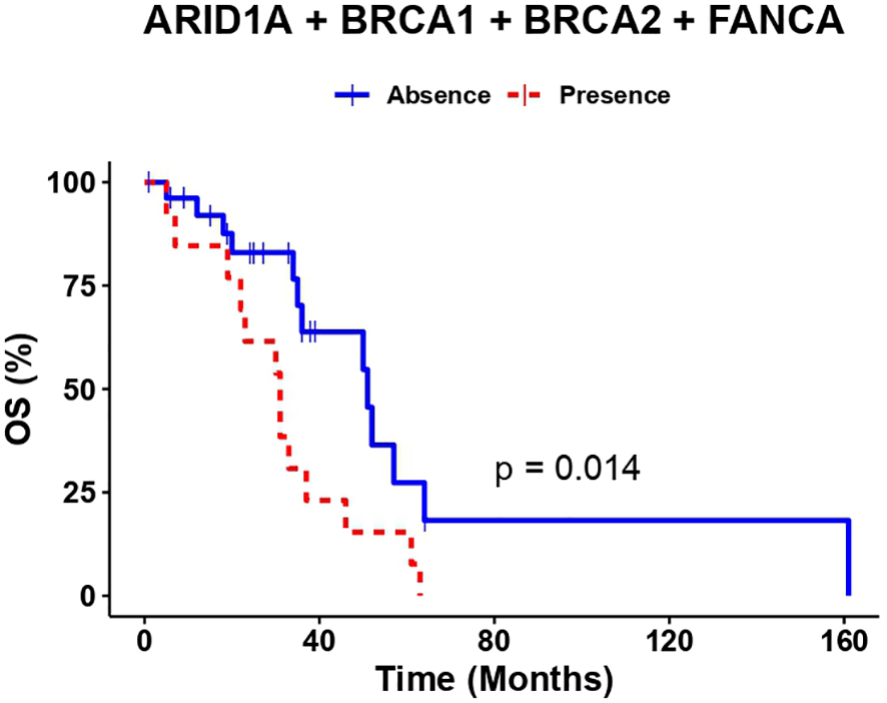
The presence of at least one variant in *ARID1A*, *BRCA1*, *BRCA2 or FANCA* genes correlates with lower OS.

An overall survival analysis was performed considering the patients included with a median follow-up of 5 years (1-13 years). The median PFS of all patients was 6 months (0-45) and median OS was 34 months (1-161). An univariate and multivariate Cox analysis were performed examining the association of the individual variants, with the clinical outcomes (PFS and OS), nevertheless, no significant association was observed (data not shown). So, we considered the genes with highest number of variants observed at the multivariate analysis combined (*ARID1A*, *BRCA1*, *BRCA2* and *FANC* genes*)* and regrouped the variation frequencies of these genes into a single binary variant where at least one variation needed to be met. To assess the association between the combination of these variants and clinical outcome, we compared PFS and OS between patient’s with or without variants in those genes. Median PFS was 8 months in patients without sequence variants in selected genes, versus 4 months in patients with at least one variant of the genes selected (p-value=0.0025) (**Figure 2**). Besides, the median OS in patients without variants was 51 months versus 31 months for patients with variants of the genes selected (p-value= 0.014) (**Figure 3**).

A univariate and multivariate analysis was performed comparing the presence/absence of pathogenic variants in at least one selected gene and clinical outcome (PFS and OS). At PFS Cox models, the presence of at least one pathogenic variant, demonstrated to be predictors of PFS (univariate, HR: 3.3 (95% CI 1.5-7.5), p-value=0.004) (multivariate, HR: 3.74 (95% CI 1.44-9.74), p-value=0.006). Additionally, at OS Cox models, the presence of at least one pathogenic variant, also demonstrated to be predictors of OS (univariate, HR: 2.7 (95% CI 1.2-6), p-value=0.017) (multivariate HR: 2.87 (95% CI 1.07-7.67), p-value=0.035) (**Table 4**).

**Table 4:** Univariate and Multivariate analysis of clinical characteristics and variants in grouped genes (*ARID1A + BRCA1 + BRCA2 + FANCA)* in the analyzed population.

	PFS				OS			
	Univariate		Multivariate		Univariate		Multivariate	
Characteristic	HR (IC 95%)	p-value	HR (IC 95%)	p-value	HR (IC 95%)	p-value	HR (IC 95%)	p-value
**Histology** (Ductual vs Lobulillar)	1.1 (0.39-3.3)	0.82			0.71 (0.24-2.1)	0.54		
Molecular classification* (Luminal A vs Luminal B vs HER enriched vs TNBC)	0.88 (0.61-1.3)	0.51	0.53 (0.03-9.18)	0.669	1.3 (0.81-2)	0.3	0.50 (0.02-9.77)	0.651
**Estrogens** (Negative vs Positive)	1.1 (0.47-2.7)	0.79	1.02 (0.00-106.)	0.992	0.4 (0.15-1.1)	0.067	0.29 (0.00-31.9)	0.612
**Progesterone** (Negative vs Positive)	0.71 (0.31-1.7)	0.43	0.23 (0.02-2.13)	0.197	0.4 (0.16-1)	0.051	0.22 (0.02-2.46)	0.223
**HER2** (Negative vs Positive)	0.5 (0.19-1.3)	0.17	1.54 (0.09-24.8)	0.76	0.53 (0.18-1.5)	0.25	1.48 (0.08-27.6)	0.789
**KI67** (Low vs High)	1 (0.98-1)	0.93	0.99 (0.97-1.02)	0.714	1 (0.99-1)	0.58	0.99 (0.96-1.01)	0.422
**Oncologic history** No vs Yes	0.7 (0.31-1.6)	0.39			0.46 (0.2-1.1)	0.076		
Chemotherapy regime* (Antimitotic vs Hormone therapy vs Alkylating-antimitotic vs Alkylating)	0.94 (0.78-1.1)	0.47	0.85 (0.69-1.04)	0.116	0.99 (0.83-1.2)	0.94	0.85 (0.68-1.07)	0.177
**Radiotherapy** (Negative vs Positive)	0.67 (0.29-1.5)	0.35			0.57 (0.24-1.4)	0.2		
**ARID1A + BRCA1 + BRCA2 + FANCA** (Absent vs Present)	3.3 (1.5-7.5)	**0.004**	3.74 (1.44-9.74)	**0.006**	2.7 (1.2-6)	**0.017**	2.87 (1.07-7.67)	**0.035**

*All mentioned characteristics were compared in the statistical analysis. The bold values denote statistically significant P values.

## 4 Discussion

Variants in DNA damage response (DDR) genes in cancer are important biomarkers for treatment selection and are also functionally important, since malfunctioning DDR can potentially increase genomic instability, eventually leading to treatment resistance or relapse (Reviewed in 28). Here we analyzed a 40-patient cohort of metastatic breast cancer patients in search for variations in DDR genes and found 13 previously identified variants and 9 that had not been reported before. These findings contribute to the understanding of the genomic landscape of metastatic breast cancer in the Mexican-mestizo population which, due to its diverse ancestry (29), is likely to differ from the mostly Caucasian populations of North America (30) and Europe (31) where the genomic characterizations of metastatic breast cancer has been reported.

Sequence variants in *TP53* accounted for roughly half of our findings. A similar proportion was previously reported in metastatic tumors (31) but in breast tumors in general, *TP53* variants accounted for less than 10% (32), highlighting the high risk of metastasis associated with *TP53* variants. Half (5/10) of the variants that we report here had not been reported before, suggesting they are exclusive or more frequent in the Mexican-mestizo population and underlining the importance of studying local populations.

Variants in *PALB2* are usually reported in frequencies around 1%, second to the BRCA genes at 3-5% (6, 33, 34). The absence of is *PALB2* variants in our cohort is similar to the low incidence observed in a separate study where only two of 115 patients carried *PALB2* variants (35), suggesting that *PALB2* variants are scarce in the Mexican-mestizo population.

We found a significant association between the presence of at least one pathogenic variant and worse PFS and OS. Three of these genes are related to the DNA Interstrand Crosslink Repair: BRCA1 and BRCA2 are the quintessential breast cancer susceptibility genes (36). *FANCA* variations are the most frequent in Fanconi anemia (37) and, according to recent reports, it might be the only *FANC* gene involved in hereditary cancer (38). These findings bolster previous reports on the association between variations in individual genes of the DNA Interstrand Crosslink Repair pathway and breast cancer susceptibility in Iranian (39) and Chinese populations (40). Interestingly, while there is evidence that variations in the DDR pathways –particularly HR– sensitize several cancer types to chemotherapy (15), secondary variants in these genes can generate resistance to alkylating agent therapy (41, 42). These findings suggest that the variants in *BRCA1, BRCA2*, and *FANCA*, associated with worse prognosis in our sample set, might have contributed to chemotherapy resistance. The mechanisms underlying this phenomenon will undoubtedly motivate further analysis. Sequence variants in the fourth gene, *ARID1A*, have been associated with breast cancer brain metastasis, though the specific variants that we found had not been reported before (14).

In Latin America, breast cancer is detected late in its development (21) a trend that can only be reverted through optimized screening strategies. Since tumors with altered *ARID1A* are sensible to PARP inhibitors (43) and its variants are frequent in cohorts as small as the 40 patients that we report here, we suggest screening tumors for variants *ARID1A* in the Mexican-mestizo population. Such screening might broaden the treatment options for breast cancer patients, as these variants have been associated with enhanced effects of treatments such as ATR inhibitors and Gemcitabine in ovarian cancer (44). Further studies will confirm whether the high prevalence of *ARID1A* variants in tumor samples is valid for other Latin American populations and whether there is a functional relationship between *ARID1A* and the DNA Interstrand Crosslink Repair genes. Additionally, whether the *ARID1A* variants we observed were acquired during tumor development or already present in the germline and thus related to cancer susceptibility besides response to treatment remains to be determines. If these variants are germline, *ARID1A* might be a better indicator of cancer risk than PALB2 in the Mexican-mestizo populations.

Our study was limited by the number of genes sequenced and the relatively low number of samples; additionally, the sequencing was performed only from tumor. A larger sample would provide a more comprehensive perspective of the variations in these and other genes; however, our sample included only metastatic breast cancer patients, which represent less than 20% of the total breast cancer cases (45, 46).

## 5 Conclusions

Our findings contribute to the description of the sequence variation landscape of metastatic breast cancer in the Mexican-mestizo population. We found the expected high frequency variants in TP53 and *BRCA 1* and *2*; conversely, *PALB2* variants seem scarce compared to other reported populations. The presence of at least one pathogenic variant in the *ARID1A, BRCA1, BRCA2*, or *FANCA* genes remains predictor of worse progression-free survival and overall survival.

## Data availability statement

All data generated or analyzed during this study are included in this published article and are available from the corresponding author on reasonable request.

## Ethics statement

The studies involving human participants were reviewed and approved by National Cancer Institute of México, Ethics and Scientific Committee. The patients/participants provided their written informed consent to participate in this study.

## Author contributions

Conceptualization, CP-P, DC and RV-R; Data curation, AM-G; Formal analysis, OM-C, AC-P, RV-R and AA-M; Investigation, DC; Methodology, ER-G, OM-C, MG-E and NJ-H; Resources, CL-C; Supervision, DC and CP-P; Writing – original draft, CP-P; Writing – review and editing, EL-U and CP-P. All authors contributed to the article and approved the submitted version.
