# Supplementary material for: Pathogenic variant profile in DNA damage response genes correlates with metastatic breast cancer progression-free survival in a Mexican-mestizo population
Source: Front Oncol. 2023 Apr 27;13:1146008. doi: 10.3389/fonc.2023.1146008 (PMC10174330; doi:10.3389/fonc.2023.1146008)
Supplement: Supplementary file 1 [file Table_1.docx]

Supplementary

| Genes | Target CDS + UTR (pb) | Covered (%) |
| --- | --- | --- |
| ATM | 13 777 | 99.5 |
| CHK2 | 2 271 | 99.82 |
| FANCA | 6 625 | 97.52 |
| FANCB | 3 108 | 100 |
| FANCC | 6 008 | 99.23 |
| FANCD2 | 6 387 | 95.93 |
| PARP-1 | 4 610 | 99.54 |
| PALB2 | 4 188 | 100 |
| RAD50 | 6 847 | 99.8 |
| RAD51 | 2 530 | 99.2 |
| TP53 | 3 208 | 98.85 |
| ARID1A | 8 785 | 99.55 |
